# Evaluating the readability of recruitment materials in veterinary clinical research

**DOI:** 10.1111/jvim.16857

**Published:** 2023-09-27

**Authors:** Charly McKenna, Mindy Quigley, Tracy L. Webb

**Affiliations:** ^1^ Ontario Veterinary College University of Guelph Guelph Ontario Canada; ^2^ Virginia‐Maryland College of Veterinary Medicine Virginia Tech Blacksburg Virginia USA; ^3^ Department of Clinical Sciences Colorado State University Fort Collins Colorado USA

**Keywords:** client‐owned animal, clinical trials, companion animal, Flesch Reading Ease Score, Flesch‐Kincaid Grade Level

## Abstract

**Background:**

Owner comprehension is vital to recruitment and study success, but limited information exists regarding the readability of public‐facing veterinary clinical trial descriptions.

**Objectives:**

The current study sought to evaluate the readability of public‐facing online veterinary clinical trial descriptions from academic institutions and private referral practices.

**Animals:**

None.

**Methods:**

This prospective study assessed readability in a convenience sample of veterinary clinical trial study descriptions using 3 common methods: the Flesch‐Kincaid Grade Level (F‐K), Flesch Reading Ease Score (FRES), and online Automatic Readability Checker (ARC). Results were compared across specialties and between academic and private institutions.

**Results:**

Grade level and readability consensus scores (RCSs) were obtained for 61 online clinical trial descriptions at universities (n = 49) and private practices (n = 12). Average grade‐level RCS for study descriptions was 14.13 (range, 9‐21). Using Microsoft Word, the FRES score was higher in descriptions from universities compared to private practices (*P* = .03), and F‐K scores were lower in university compared to private practice descriptions (*P* = .03). FRES (*P* = .07), F‐K (*P* = .12), and readability consensus (*P* = .17) scores obtained from ARC were not different between institution types. Forty‐eight studies (79%) had RCSs over 12, equivalent to reading material at college or graduate school levels.

**Conclusions and Clinical Importance:**

Similar to other areas in veterinary communication, the evaluated veterinary clinical trial descriptions used for advertising and recruitment far exceeded the recommended 6th‐grade reading level for medical information. Readability assessments are straightforward to conduct, and ensuring health literacy should be a customary best practice in veterinary medicine and clinical research.

**Abbreviations**
AAHSD: American Veterinary Medical Association Animal Health Studies Database
AMA: American Medical Association
ARC: Automatic Readability Checker
CRO: clinical research organization
F‐K: Flesch‐Kincaid
FRES: Flesch Reading Ease Score
NIH: National Institutes of Health
RCS: Readability Consensus Score

## 1 INTRODUCTION

Written health communications can be assigned a United States (US) educational grade level after assessment of variables such as sentence length and number of syllables using the Flesch‐Kincaid (F‐K) Grade Level and Flesch Reading Ease Score (FRES).^1^, ^2^ The average American can read at an 8th‐grade level, but the recommendations for written health information from the National Institutes of Health (NIH) and the American Medical Association (AMA) are lower, with a suggested target between the 4th and 6th grade levels.^3^, ^4^ This recommendation is evidence‐based, as low health literacy is correlated with negative health outcomes.^3^ The use of a Health Literacy Model is commonplace in human medicine and is designed to support 3 main components that are essential to the concept of informed consent: “read, understand, and act.” “Read” refers to functional literacy that provides information at a level that the reader can understand. “Understand,” or communicative literacy, enables the reader to discuss the provided information. “Act” refers to critical literacy, or the ability of the reader to use the provided information to make decisions. Informed consent is an essential component of the practice of both quality clinical medicine and research.^5^, ^6^

In both human and veterinary clinical trials, failure to meet recruitment goals is the single largest driver of delays in study completion, cost overruns, and excessive workloads, and is a serious threat to study validity and the integrity of evidence‐based medicine.^7^, ^8^, ^9^ A lack of understanding can have serious ramifications for studies, patients, and the practice of medicine. It can reduce patient recruitment, and thereby inhibit access to cutting edge therapies, as well as affecting patient retention and compliance with study directives. In veterinary practice and clinical research, the pet owner provides consent for animals participating in research studies. Unlike in human medicine, there are no established guidelines for handouts or other clinical trial descriptions and information provided to pet owners in veterinary medicine. Literature evaluating the concept of “readability” in public‐facing veterinary clinical trial‐related information is scarce. Readability, for veterinary purposes, is an objective measure defined according to the ease with which a pet owner can read and comprehend written health information. Clinical trial consent forms from multiple veterinary study centers have been evaluated for readability, with all of the included forms (n = 53) outside of the NIH‐ and AMA‐recommended 6th grade reading level.^10^ Explanatory language used in consent forms is often shared with recruitment and study advertising.

The objective of the current study was to evaluate the readability of public‐facing online veterinary clinical trial descriptions from both academic institutions and private practices. We hypothesized that the collected recruitment materials would be scored above the average American's reading level and would also exceed the NIH‐ and AMA‐recommended 6th grade reading level for health information.

## 2 MATERIALS AND METHODS

### 2.1 Study design

This study was a review of currently‐available, public‐facing veterinary clinical trial recruitment materials.

### 2.2 Text acquisition

A convenience sample of project descriptions was obtained and processed by a single investigator (MQ) between May 12, 2022 and July 7, 2022 from the American Veterinary Medical Association Animal Health Studies Database (AAHSD), university clinical trials websites, and clinical research organization (CRO) websites. Project descriptions were selected to ensure a broad representation of disciplines and institution types. To be included in the study, the description had to contain at least 100 words because the online readability tool utilized is unable to generate accurate scores from shorter abstracts. Each study description was copied from the public‐facing website and pasted into a Microsoft Word document for further analysis. If the study description was of insufficient length to yield a readability score, additional explanatory text was copied from background/additional information sections, if available. Descriptions with insufficient text for analysis were excluded.

### 2.3 Readability assessment

Readability of each study description was assessed using 3 methods: the Automatic Readability Checker (ARC; a free, web‐based readability tool: https://readabilityformulas.com/free-readability-formula-tests.php) and Microsoft Word F‐K Grade Level and FRES tools. Study descriptions were each copied into the web‐based ARC, which uses 7 readability formulas (Table 1) to assign a Readability Consensus Score (RCS) with reading and grade level. The ARC output value (range: US Kindergarten‐College grade levels, represented numerically 0‐12+) is an average based on the 7 tests. Lower scores are associated with written material that is more easily read and understood. Using Microsoft Word, FRES and F‐K scores were obtained from the collected study descriptions.^10^ The calculation formulas used in Microsoft Word are included in Table 1. The F‐K values correspond to the US grade levels at which a piece of written material is expected to be read and understood. FRES values range from 0 to 100 with higher scores denoting easier reading material, with a FRES score of 60 corresponding to a 6th‐grade reading level.

**TABLE 1:** Readability test formulas used by the web‐based Automatic Readability Checker (ARC) and Microsoft Word.

Methodology	Readability test	Formula
ARC	Flesch Reading Ease	206.835 − (1.015 × ASL) − (84.6 × ASW)
	Flesch Grade Level	(0.39 × ASL) + (11.8 × ASW) − 15.59
	Gunning's Fog Index	0.4 (ASL + PHW)
	Simple Measure of Gobbledygook (SMOG)	3 + Square Root of Polysyllable Count
	Coleman‐Liau Index	0.0588 × 448 (average number of letters per 100 words) − 0.296 × 4.0 (average number of sentences per 100 words) − 15.8
	Automated Readability Index	4.71 (characters/words) + 0.5 (words/sentences) – 21.43
	Linsear Write	Text based^a^
Microsoft Word	Flesch–Kincaid Grade Level	(0.39 × ASL) + (11.8 × ASW) − 15:59
	Flesch Reading Ease Score	206.835 − (1.015 × ASL) − (84.6 × ASW)

*Note*: Character = any letter or number; ASL = Average Sentence Length (ie, the number of words divided by the number of sentences); ASW = Average number of Syllable per Word (ie, the number of syllables divided by the number of words); PHW = Percentage of Hard Words.

^a^ https://readabilityformulas.com/free‐readability‐formula‐tests.php.

### 2.4 Statistical evaluation

Descriptive statistics were performed using Microsoft Excel. The continuous data were described using means and SD and analyzed using a *t* test if normality was met. If normality was not met, a Wilcoxon 2 sample test was used to compare the groups. A *P*‐value of .05 was used to determine statistical significance. SAS v9.4 (SAS Institute Inc., Cary, North Carolina) was used to perform all analyses. Agreement between 2 scores was evaluated using Intraclass correlation coefficient (ICC) and compared visually using a Bland‐Altman plot. This analysis was performed using MedCalc Statistical Software version 20.218 (MedCalc Software Ltd, Ostend, Belgium).

## 3 RESULTS

### 3.1 Study descriptions

Grade level and RCSs were obtained for a total of 61 clinical research study descriptions available between May 12, 2022 and July 7, 2022. Study listings were collected from the AAHSD (n = 30, 49%), university clinical trial websites (n = 26, 43%), and private referral practice websites (n = 5, 8%). Studies were associated with the following medical specialties: cardiology (n = 5), clinical pathology (n = 2), dermatology (n = 5), genetics (n = 1), internal medicine (n = 12), large animal (n = 3), neurology (n = 4), oncology (n = 22), rehabilitation (n = 1), and surgery (n = 6). Forty‐nine studies (49/61, 80%) were conducted primarily at universities, whereas 12 studies (12/61, 20%) took place primarily at private practices.

### 3.2 Reading level results

**FIGURE 1:**
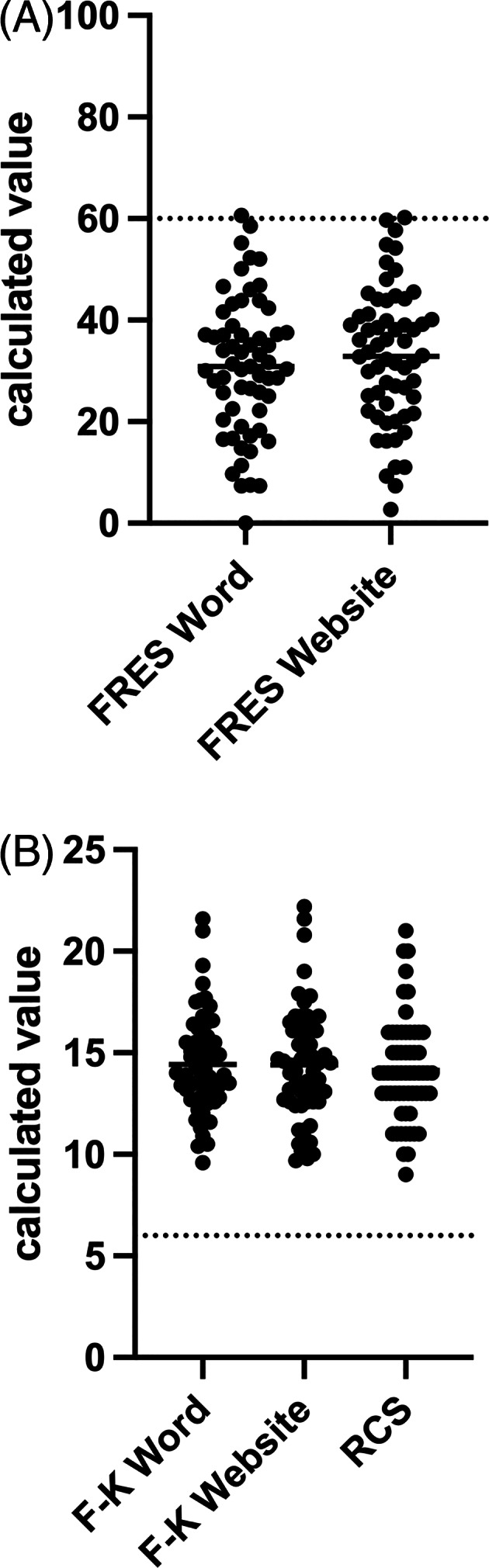
Readability assessment results for 61 veterinary clinical trial descriptions. Individual and mean values are represented. F‐K, Flesch–Kincaid; FRES, Flesch Reading Ease Score; RCS, Readability Consensus Score; Word, Microsoft Word; Website, Automatic Readability Checker (https://readabilityformulas.com/free‐readability‐formula‐tests.php). Dotted line represents 6th grade reading level: (A) 60 for FRES, with higher values being more readable, and (B) 6 for F‐K and RCS, with lower levels being more readable.

The average ARC RCS for all study descriptions was 14.13 (range, 9‐21). Thirteen studies (21%) had an ARC RCS of 12 or under, that is, equivalent to a high school reading level. The average FRES obtained from Microsoft Word was 30.91 (range, 0‐60.6), whereas the ARC yielded an average FRES of 32.91 (range, 2.7‐60.2). A single study (1.6%) had the recommended target FRES of 60 or higher. For F‐K Grade Level scores, Microsoft Word yielded an average F‐K value of 14.42 (range, 9.6‐21.6), whereas the ARC average was 14.40 (range, 9.7‐22.2). No studies had the average American reading level or the NIH/AMA recommended reading level (Figure 1).

### 3.3 Comparisons between groups

Due to low study numbers in several disciplines, readability comparisons could only be performed between oncology (n = 22) and internal medicine (n = 12); there were no significant differences between the 2 groups for any of the evaluated readability scoring methods (*P* > .19). The FRES score obtained from Microsoft Word was statistically significantly higher in listings from universities (n = 49, mean 32.70, range, 7.4‐60.6) compared to private practices (n = 12, mean 23.57, range, 0‐43.2) (*P* = .03). The F‐K scores obtained from Microsoft Word were statistically significantly lower in university (mean 14.10, range, 9.6‐21) compared to private practice listings (mean 15.77, range, 12.8‐21.6) (*P* = .03). FRES (*P* = .07), F‐K (*P* = .12), and readability consensus (*P* = .17) scores obtained from ARC were not significantly different between institution types. Retrieval source, that is, AAHSD (n = 30) vs institutional websites (n = 31), was not significantly different in any of the measured readability scores (*P* > .21).

### 3.4 Agreement between methods

Despite the variable results, there was substantial agreement between F‐K scores obtained from Microsoft Word and ARC when an overall analysis was performed (ICC 0.93; mean of 0 on Bland‐Altman plot); agreement was slightly less for FRES scores (ICC 0.89; mean of −2.0 on Bland‐Altman plot).

## 4 DISCUSSION

All evaluated study descriptions scored above the NIH‐ and AMA‐recommended 6th‐grade reading level for health information materials and above the average American's reading level (eg, 8th grade). Based on the assessment using the ARC RCS, Microsoft F‐K grade level and Microsoft FRES, collected clinical trial recruitment materials were written at a level that would require post‐secondary education (ie, veterinary professionals) and were therefore not readily comprehensible to the general public and the average pet owner. These results are similar to previous studies on both pet owner‐focused educational handouts and study informed consent documents wherein 90%‐100% of materials were above the recommended reading level.^10^, ^11^, ^12^, ^13^

The Internet has become one of the most popular sources of health information, with 59%‐80% of adults using the internet to search for health‐related questions for themselves or their pets.^14^, ^15^, ^16^ The study descriptions selected for this study were intended to represent the average owner's experience when searching for veterinary clinical study information online, presuming that most owners search using Google or by seeking out the websites of their regional veterinary college or veterinary specialty clinics. Although the readability level of veterinary clinical study descriptions from universities was statistically significantly higher than those from private practices in 2 of the scores, the difference in readability was less than 2 grade levels and for both groups was well above recommended levels. These results support a recommendation and imperative for all types of veterinary practices and websites posting veterinary clinical trial information to employ strategies to improve readability of their written clinical study descriptions.

Of adults (n = 183) participating in a human oncology clinical research study provided with simplified and standard consent forms at 7th‐ and 16th‐grade reading levels, respectively, 62% of participants preferred the simplified form, and 97% found it easier to read than the standard form.^17^ Importantly, the degree of understanding was the same between the simplified and standard forms, demonstrating that eliminating jargon and scientific detail did not reduce participant understanding.^17^ Additionally, a participant's overall comprehension of a study can influence the entire research process. Human patients who dropped out of a trial early are twice as likely to attribute their lack of follow‐through to a failure in understanding the informed consent than those who remain in the study.^17^ Understanding a study has a significant effect on patient anxiety and participant satisfaction.^18^ Importantly, stress has many physiological effects on an individual, ranging from mild to life‐threatening, such that stress can become a confounding factor and adversely affect research outcomes.^19^, ^20^ Employing methods to reduce stress, such as increasing feelings of control or predictability, has been proposed as the best way to reduce negative physiological effects of “difficult‐to‐manage stressors” in research animals.^20^ Improved understanding of processes and procedures is one method of increasing feelings of control and predictability for pet owners and their pets.

Clear, thorough, and trustworthy communication is similarly necessary for the majority of pet owners to comply with veterinary recommendations. Failure to provide accessible information compromises the ability of pet owners to make informed medical decisions and might lead to dissatisfaction with and mistrust of veterinary professionals.^12^ When associated with voluntary participation in veterinary clinical research, lack of accessible information can decrease study quality and thereby inhibit advancements in veterinary medicine and translational research.

This study confirms the continued critical need to improve the readability of veterinary‐pet owner written communication in all areas.^10^, ^11^, ^12^, ^13^, ^21^, ^22^ The 3 methods used to calculate readability in this study were found to have good agreement, therefore using any available tool to measure and improve readability is likely to be helpful. Considering that mean scores correlated to “difficult” or “very difficult to read” and “best understood by college graduates,” any improvement in readability is likely to increase understanding. To encourage improved readability, websites listing veterinary and human clinical trials information could provide recommendations, resources (Table S1), and algorithms to measure readability during the posting process.

Overall, veterinary clinical trials would benefit by giving more forethought to the importance of recruitment‐related communication and encouraging a relationship‐centered approach. Advertising materials are often the first introduction to a study for participants and should be written in an active voice to promote enthusiasm about a study and create an opportunity for owners to be empowered advocates for their pets. Adhering to a modified “read, understand, and act” health literacy model, communication strategies should be at the forefront of study design, with study advertising materials calibrated for the general public. Easy‐to‐read study descriptions should employ short sentences (≤10 words), limit words with more than 3 syllables, minimize medical abbreviations, and eliminate medical jargon. Such emphasis would help improve accessibility and equity in study enrollment. In addition to readability, veterinary clinical trial recruitment materials would benefit from more focus on animal and pet‐owner factors such as the practicalities, costs, and logistics of participation. Continued training in science communication can be an invaluable tool for researchers and should be utilized to increase participant/owner understanding of the risks and benefits of clinical trials and thereby increase ethical enrollment.

Limitations to this study include the removal of text organization (bullet points, subheadings) from the readability analyses as, generally, these make a document easier to read. In addition, only text‐based descriptions were included in this study, and recruitment materials that included visual elements such as illustrations, photos, videos, or white space in addition to written text were not included. Descriptions less than 100 words could not be included in the analysis because of readability determination method limitations. The online readability checker used in this study gives an error message if the sample is less than 100 words, and the instructions state that a sufficient sample is between 200 and 600 words. A recent study evaluating the consistency of the best‐known readability equations found results to be more consistent with increasing length of the passage examined up to 900 words.^23^ Additionally, some group sizes were too small to allow comparisons, however, the available data do not suggest that any specific group is currently achieving recommended reading levels.

Similar to other areas in veterinary communication, evaluated veterinary clinical study descriptions used for advertising and recruitment far exceeded the recommended 6th‐grade reading level for medical information. As readability assessments are straightforward to conduct, ensuring health literacy should become a customary best practice in veterinary medicine and veterinary clinical research. Empowering all pet owners to “read, understand, and act” through clear, accessible, and trustworthy communication accelerates advancements in veterinary medicine by creating partners in veterinary care and committed participants in veterinary clinical research. Efforts to encourage readability can greatly improve current practices. Future studies should evaluate the effect of incorporating improved readability and multi‐lateral communication strategies on trial recruitment and outcomes.

## CONFLICT OF INTEREST DECLARATION

Authors declare no conflict of interest.

## OFF‐LABEL ANTIMICROBIAL DECLARATION

Authors declare no off‐label use of antimicrobials.

## INSTITUTIONAL ANIMAL CARE AND USE COMMITTEE (IACUC) OR OTHER APPROVAL DECLARATION

Authors declare no IACUC or other approval was needed.

## HUMAN ETHICS APPROVAL DECLARATION

Authors declare human ethics approval was not needed for this study.

## Supporting information


**Table S1:** Resources for improving readability.
