# Supplementary material for: Evaluating the readability of recruitment materials in veterinary clinical research
Source: J Vet Intern Med. 2023 Sep 27;37(6):2125–30. doi: 10.1111/jvim.16857 (PMC10658532; doi:10.1111/jvim.16857)
Supplement: Supplementary file 1 — Table S1: Resources for improving readability. [file JVIM-37-2125-s001.pdf]

Supplemental Table 1: Resources for improving readability.

US Food and Drug Administration (FDA) Information Sheet: A Guide for Informed Consent - Guidance for Institutional Review Boards and Clinical Investigators (January 1998)	https://www.fda.gov/regulatory-information/search-fda-guidance-documents/guide-informed-consent
FDA: Informed Consent for Clinical Trials	https://www.fda.gov/patients/clinical-trials-what-patients-need-know/informed-consent-clinical-trials
National Institutes of Health (NIH) Search for "readability"	https://search.nih.gov/search?affiliate=nih&query=readability
Stanford University Definitions and Lay Glossary of Medical Terms	https://researchcompliance.stanford.edu/panels/hs/for-researchers/definitions
National Comprehensive Cancer Network (NCCN) Informed Consent Language (ICL) Database	https://www.nccn.org/education-research/nccn-oncology-research-program/informed-consent-language-database
